# Lentiviral vectors can be used for full-length dystrophin gene therapy

**DOI:** 10.1038/s41598-017-00152-5

**Published:** 2017-03-06

**Authors:** John R. Counsell, Zeinab Asgarian, Jinhong Meng, Veronica Ferrer, Conrad A. Vink, Steven J. Howe, Simon N. Waddington, Adrian J. Thrasher, Francesco Muntoni, Jennifer E. Morgan, Olivier Danos

**Affiliations:** 1 The Dubowitz Neuromuscular Centre, Molecular Neurosciences Section, Developmental Neurosciences Programme, UCL Great Ormond Street Institute of Child Health, 30 Guilford Street, London, WC1N 1EH UK; 2 UCL Cancer Institute, Paul O ‘Gorman Building, University College London, 72 Huntley Street, London, WC1E 6BT UK; 3 Molecular and Cellular Immunology, Institute of Child Health, University College London, 30 Guilford Street, London, WC1N 1EH UK; 4 Gene Transfer Technology Group, Institute for Womens Health, University College London, 86-96 Chenies Mews, London UK; 5 MRC Antiviral Gene Therapy Research Unit, Faculty of Health Sciences, University of the Witswatersrand, Johannesburg, South Africa; 6 Biogen, 14 Cambridge Center, Cambridge, MA 02142 USA

## Abstract

Duchenne Muscular Dystrophy (DMD) is caused by a lack of dystrophin expression in patient muscle fibres. Current DMD gene therapy strategies rely on the expression of internally deleted forms of dystrophin, missing important functional domains. Viral gene transfer of full-length dystrophin could restore wild-type functionality, although this approach is restricted by the limited capacity of recombinant viral vectors. Lentiviral vectors can package larger transgenes than adeno-associated viruses, yet lentiviral vectors remain largely unexplored for full-length dystrophin delivery. In our work, we have demonstrated that lentiviral vectors can package and deliver inserts of a similar size to dystrophin. We report a novel approach for delivering large transgenes in lentiviruses, in which we demonstrate proof-of-concept for a ‘template-switching’ lentiviral vector that harnesses recombination events during reverse-transcription. During this work, we discovered that a standard, unmodified lentiviral vector was efficient in delivering full-length dystrophin to target cells, within a total genomic load of more than 15,000 base pairs. We have demonstrated gene therapy with this vector by restoring dystrophin expression in DMD myoblasts, where dystrophin was expressed at the sarcolemma of myotubes after myogenic differentiation. Ultimately, our work demonstrates proof-of-concept that lentiviruses can be used for permanent full-length dystrophin gene therapy, which presents a significant advancement in developing an effective treatment for DMD.

## Introduction

Duchenne Muscular Dystrophy (DMD) is a severe muscle-wasting disease that arises from a lack of functional dystrophin expression in nearly all of the patient’s muscle fibres^1^. Conventional gene therapy of DMD is hindered by the large size of dystrophin cDNA (13,957 bp), which exceeds the optimal packaging capacity of commonly used viral vectors^2–4^. This presents a significant problem for the development of new DMD therapies, for which many strategies have been investigated. The delivery of truncated ‘mini-dystrophin’ transgenes to patient cells^5, 6^, exon-skipping of defective dystrophin exons^7–9^ and gene-editing of defective DMD genomes^10–13^ have all shown promise in restoring partial protein expression, however, in each case the expectation is that at best the patient phenotype could be that of a less severe form of muscular dystrophy, owing to low levels of dystrophin restoration combined with a lack of functional domains in the internally deleted dystrophin products^14^. Thus, it is important to investigate strategies for restoration of full-length dystrophin that could provide all functionality of the wild-type protein.

*Ex vivo* correction of patient stem cells has shown promise as a method for DMD gene therapy^9^. Lentiviral vectors offer a significant advantage over adeno-associated viruses (AAV) in this scenario given that AAV vectors do not commonly integrate into stem cell genomes, which leads to loss of dystrophin expression upon repeated cell divisions^15^. Furthermore, there is increasing evidence that dystrophin expression in muscle stem cells stimulates the generation of the myogenic progenitors that are required for efficient muscle regeneration^16^. Thus, lentiviruses are preferred over AAV for *ex vivo* DMD gene therapy as they enable stable transduction of a stem cell pool with a therapeutic cassette, whilst concurrently enhancing muscle stem cell functionality prior to transplantation.

Human immunodeficiency virus type 1 (HIV-1) lentiviral vectors have been widely used for delivering transgenes to dividing and non-dividing cells for gene therapy applications^17^. Lentiviral vectors are limited by their transgene-carrying capacity, which becomes increasingly inefficient as the viral genomic load exceeds 10,000 bp^2, 18, 19^. During virion assembly, lentiviruses package two copies of their single-stranded RNA genome, which is reverse-transcribed to form a double-stranded DNA provirus. Recently it has been reported that vector efficacy reduces in correlation with an increase in the size of vector RNA and that lentiviral vector integration is impeded when delivering large transgenes^19, 20^. This suggests that vector packaging limits could be circumvented by reducing the amount of RNA to be packaged into a vector particle. It is well established that during lentiviral reverse transcription, template-switching events take place as reverse transcriptase synthesises a DNA provirus from a dimeric RNA genome, occurring most frequently at homologous regions^21, 22^. When heterozygous RNA genomes are packaged into a lentivirus, template-switching events result in genetic recombination and the production of chimeric proviruses^23, 24^.

In this work, we have investigated the capacity for lentiviral vectors to deliver full-length dystrophin for DMD gene therapy. We initially profiled the packaging capacity of standard lentiviral vectors and proof-of-concept for a method designed to circumvent restrictions on the length of transgenes that can be delivered to cells, before successfully demonstrating that lentiviruses can be used to deliver full-length dystrophin to DMD myoblasts as a proof-of-concept *ex vivo* gene therapy strategy. This approach could provide full, permanent dystrophin functionality, which has been unachievable with competing gene therapy technologies.

## Results

**Figure 1:**
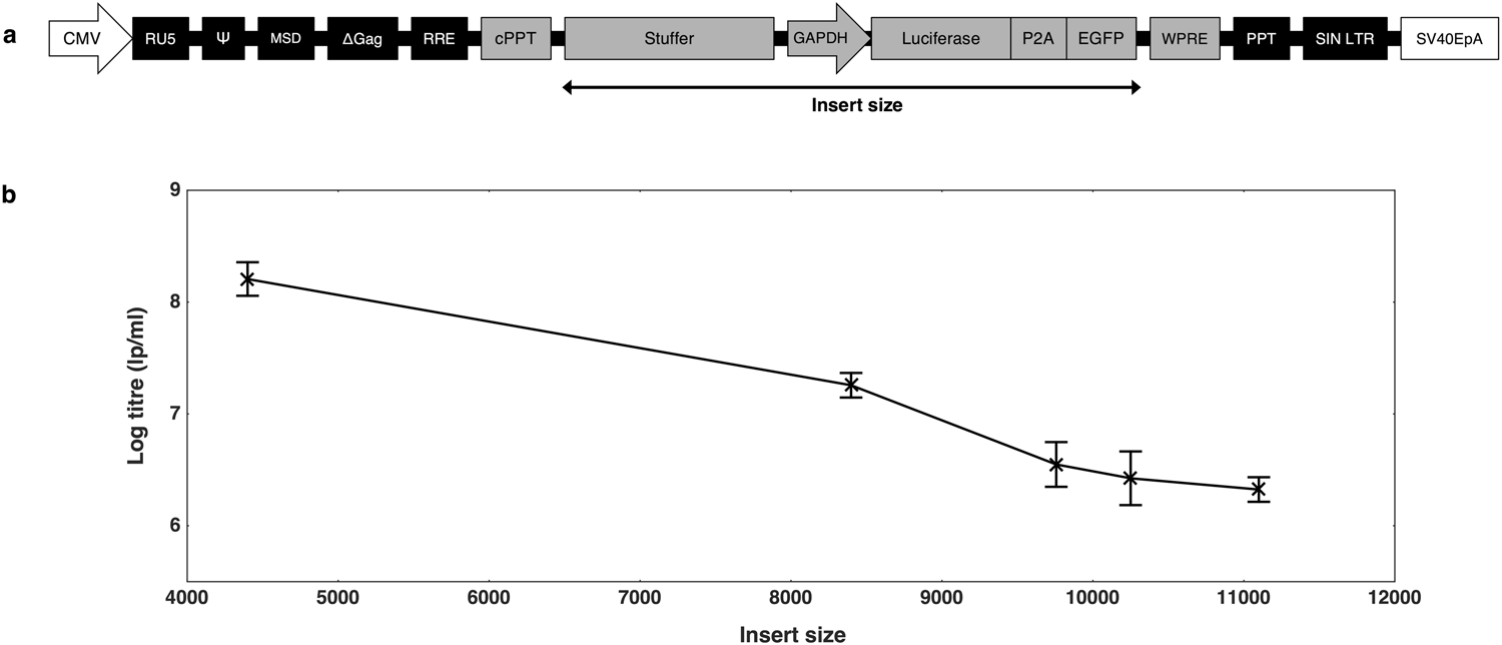
Profiling the titres of lentiviral vectors containing large inserts. (**a**) A GAPDH-Luciferase-P2A-GFP construct was modified to contain stuffers of various sizes to provide a range of insert sizes for packaging into lentiviral vectors and titre comparison by flow cytometry. The insert is regarded as all content spanning the first nucleotide of the stuffer sequence until the final nucleotide of GFP. (**b**) Vectors of various sizes were titred by GFP output on HEK 293T cells. The trend shows loss of functional titre in response to increased payload, with titres falling 2 orders of magnitude as the insert size increases from 4,400 to 11,099 base pairs. Titres are expressed as mean lentiviral particles per millilitre (lp/ml) with error bars representing standard deviation from the mean. N = 3 for all samples.

We initially set out to determine the upper-range of lentiviral transgene capacity using a standard lentiviral backbone. Varying lengths of a custom stuffer sequence were cloned upstream of a glyceraldehyde 3-phosphate dehydrogenase (GAPDH) promoter-driven lentiviral vector expressing a bicistronic Luciferase-T2A-GFP construct (Fig. 1a). This provided a range of provirus sizes for titre comparison, whilst ensuring that the content and size of the expression cassette remained constant.

As expected, titration of the stuffer constructs by green fluorescence protein (GFP) output showed that lentivirus functional titres reduce as the size of the payload increases (Fig. 1b). Interestingly, the rate of titre loss appeared to slow at the upper-range, where titres of greater than 3 × 10^6^ lp/ml were achievable with an insert in excess of 11,000 bp. Given that the dystrophin coding sequence is 11,058 base pairs, it seemed feasible that functional titres could be obtained with lentiviral vectors carrying full-length dystrophin, albeit with a reduced titre. To combat this titre loss, we sought to investigate a mechanism for rescuing gene transfer efficiency when delivering large transgenes.

### Design and development of a template-switching lentivirus for large transgene delivery

**Figure 2:**
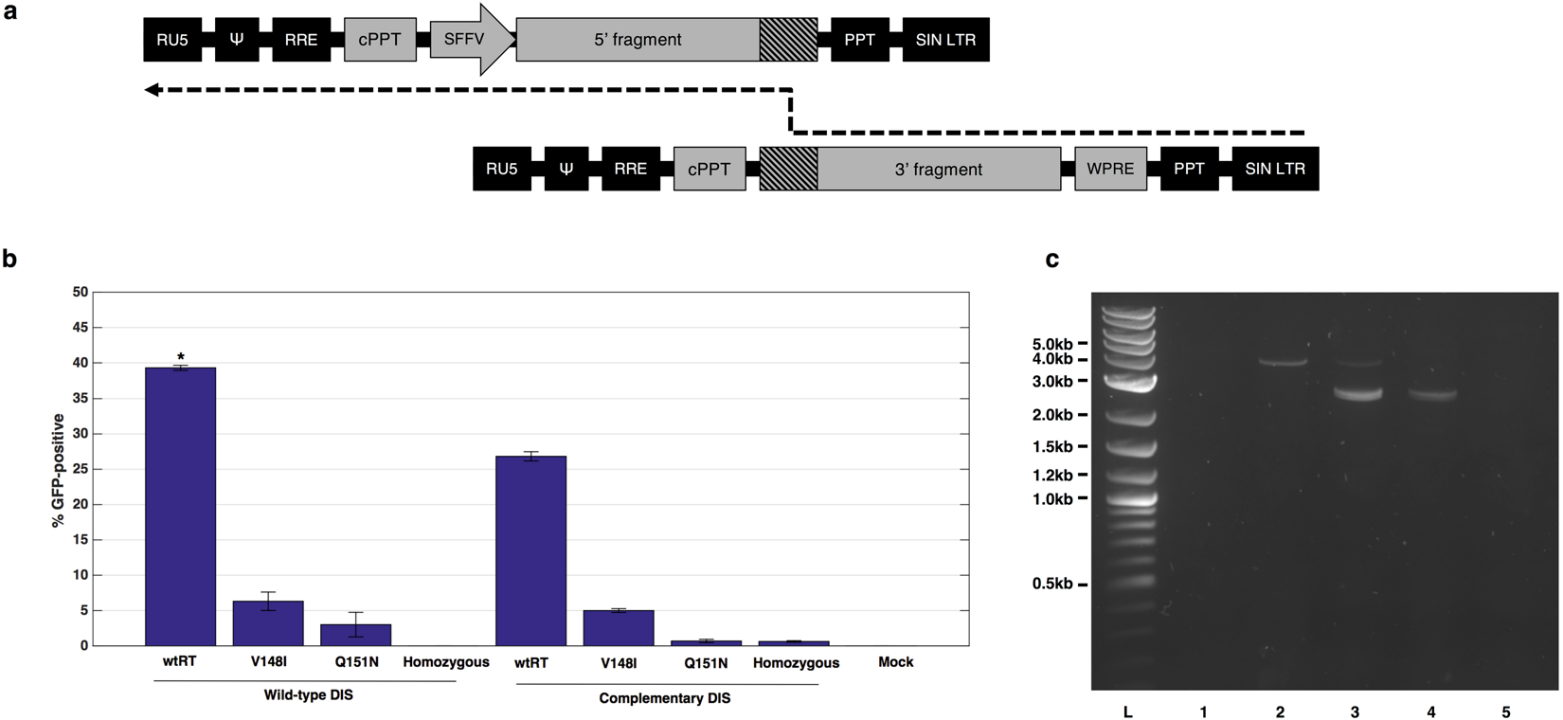
Design and development of a template-switching lentiviral vector. (**a**) Schematic representing the pathway for reconstituting a full-length provirus from a heterozygous viral genome. Reverse-transcription proceeds until reaching a region of homology where the reverse-transcription complex can undergo template-switching and reconstitute the full-length sequence. (**b**) Examination of factors affecting the rate of NeoR-IRES-GFP reconstitution. Modified lentiviruses (reverse-transcriptase mutants V148I or Q151N and/or complementary DIS) were compared to unmodified vectors. GFP output was quantified by flow cytometry, where wild-type homozygous vectors were used to set the baseline for GFP expression. All samples are N = 3. *P < 0.05 by Kruskal-Wallis test with Dunn’s post-hoc analysis. Bars represent average GFP readings and standard deviation. (**c**) PCR analysis of NIGW reconstitution in GFP-sorted HeLa cells. Separation of products on a 1% agarose gel reveals a 3.7 kb band in the full-length NIGW positive control (2) and the wtRT.wtDIS heterozygous sample (3). 2.4 kb and 2.5 kb bands were detected in the homozygous sample (4) and the heterozygous sample. All three bands were undetectable in the non-transduced (1) and water (5) control reactions. L = NEB 2-log ladder.

We investigated a novel approach for increasing lentiviral payload capacity, in which we sought to exploit the dimeric lentiviral genome and spread the full-length dystrophin sequence over two co-packaged RNA copies, with the aim of forcing recombination during reverse transcription (schematic represented in Fig. 2a). We identified template-switching and heterozygous co-packaging as the primary factors to target in engineering a chimeric provirus. Reverse transcriptase was mutated to incorporate V148I or Q151N, which template-switch more frequently than wild-type reverse transcriptase^25^. The dimer initiation signal (DIS) of the viral packaging signal was mutated to mediate Watson-Crick base pairing between corresponding RNA copies and increase the frequency of heterozygous particle formation^26–28^.

We conducted an initial investigation attempting to reconstitute a neoR-IRES-GFP-WPRE construct (NIGW) to screen the effects of our modifications and establish an optimal vector configuration. HeLa cells were transduced at a multiplicity of infection (MOI) of 40 viral copies per cell, with GFP output measured by flow cytometry 3 days after transduction (vector schematics are presented in Supplementary Figure S1).

The hetero.wtRT.wtDIS vector (containing both 5′NIGW and 3′NIGW, a wild-type reverse transcriptase and wild-type DIS) produced the strongest effect, generating 40% GFP-positive cells (P = 0.008 by Kruskal-Wallis test) (Fig. 2b). These cells were neomycin-selected and fluorescence-activated cell sorted (FACS) prior to genomic DNA extraction. PCR amplification of the provirus yielded a 3.7 kb amplicon, which matched the original full-length NIGW construct, indicating successful reconstitution of the expression cassette (Fig. 2c). Reverse transcriptase mutants and complementary DIS sequences were ineffective in improving on this level of efficiency, indicating that wild-type HIV-1 components were optimal for our strategy.

**Figure 3:**
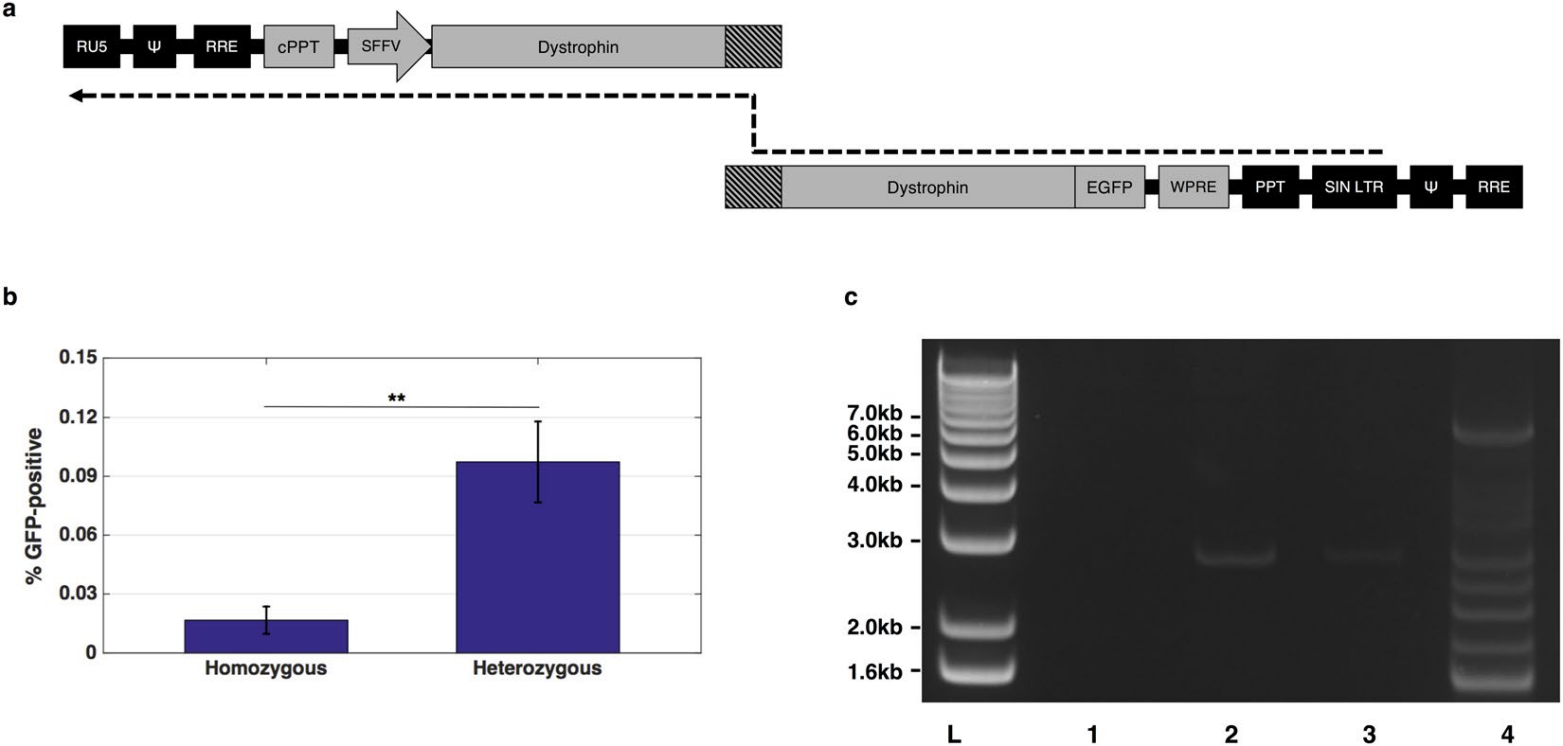
Full-length dystrophin delivery with template-switching vector. (**a**) Schematic for the modified dystrophin vector. DNA synthesis must initiate on the 5′ strand and strand transfer to the 3′ LTR will then permit synthesis of a functional provirus following template-switching within the region of dystrophin homology (shaded). A functional provirus can only be produced following recombination because essential elements are split between both strands. (**b**) Analysis of dystrophin-GFP expression in HEK 293T cells at 4 days post-transduction. Mock-transduced HEK 293T cells were used to set the baseline of GFP fluorescence. Bars represent average GFP readings and standard deviation. All samples are N = 6. **P < 0.01 by Mann-Whitney U test. (**c**) Nested PCR for full-length dystrophin-GFP from transduced HEK 293T genomic DNA. Products separated on 1% agarose gel show the presence of a 6.2 kb band in sample transduced with the heterozygous vector. L, 10 kb+ ladder; 1, water; 2, mock-transduced HEK 293T; 3, homozygous-transduced HEK 293T; 4, heterozygous-transduced HEK 293T.

A dystrophin-GFP fusion protein was employed in subsequent experiments to enable rapid detection of any full-length dystrophin expression by flow cytometry. The vector components in each corresponding RNA copy were rearranged to render the vector dependent on template-switching within dystrophin RNA and minimise expression from non-recombinants. The modified vectors were termed TS.5′DYS and TS.3′DYS to denote the template-switching dependence (Fig. 3a). Given that template-switching particles could give rise to non-functional reverse transcriptase products, qPCR titration was deemed an unsuitable method for vector titration and these vectors were instead titred by p24 ELISA.

HEK 293T cells were transduced at a dose of 400 ng p24 per 10^5^ cells. Four days after transduction, cells were harvested and analysed by flow cytometry (dot plots depicted in Supplementary Figure S3). Approximately 0.1% of cells transduced with the heterozygous vector (TS.hetero) were GFP positive (Fig. 3b), compared to the 0.016% derived from TS.homo transductions (p = 0.002 by Mann-Whitney U test, n = 6). A nested PCR from extracted genomic DNA yielded the expected 6.2 kb amplicon in TS.hetero-transduced samples, indicating the presence of a recombinant full-length dystrophin provirus (Fig. 3c). This band was absent from the homozygous control, suggesting minimal reverse-transcription of unrecombined proviruses. The 6.2 kb band was excised and subcloned for sequence analysis, which matched the corresponding region of full-length dystrophin. In the agarose gel image, it is clear that the TS.hetero PCR also produced several smaller bands. One of these bands was subcloned for sequence analysis, which revealed an internally-truncated dystrophin product, presumably generated by off-target recombination (Supplementary Figure S4). Given the low functional titre and off-target recombinants, at this stage we considered this strategy to be suboptimal for preclinical gene therapy investigations.

### Full-length dystrophin gene therapy can be achieved with standard lentiviral vectors

**Figure 4:**
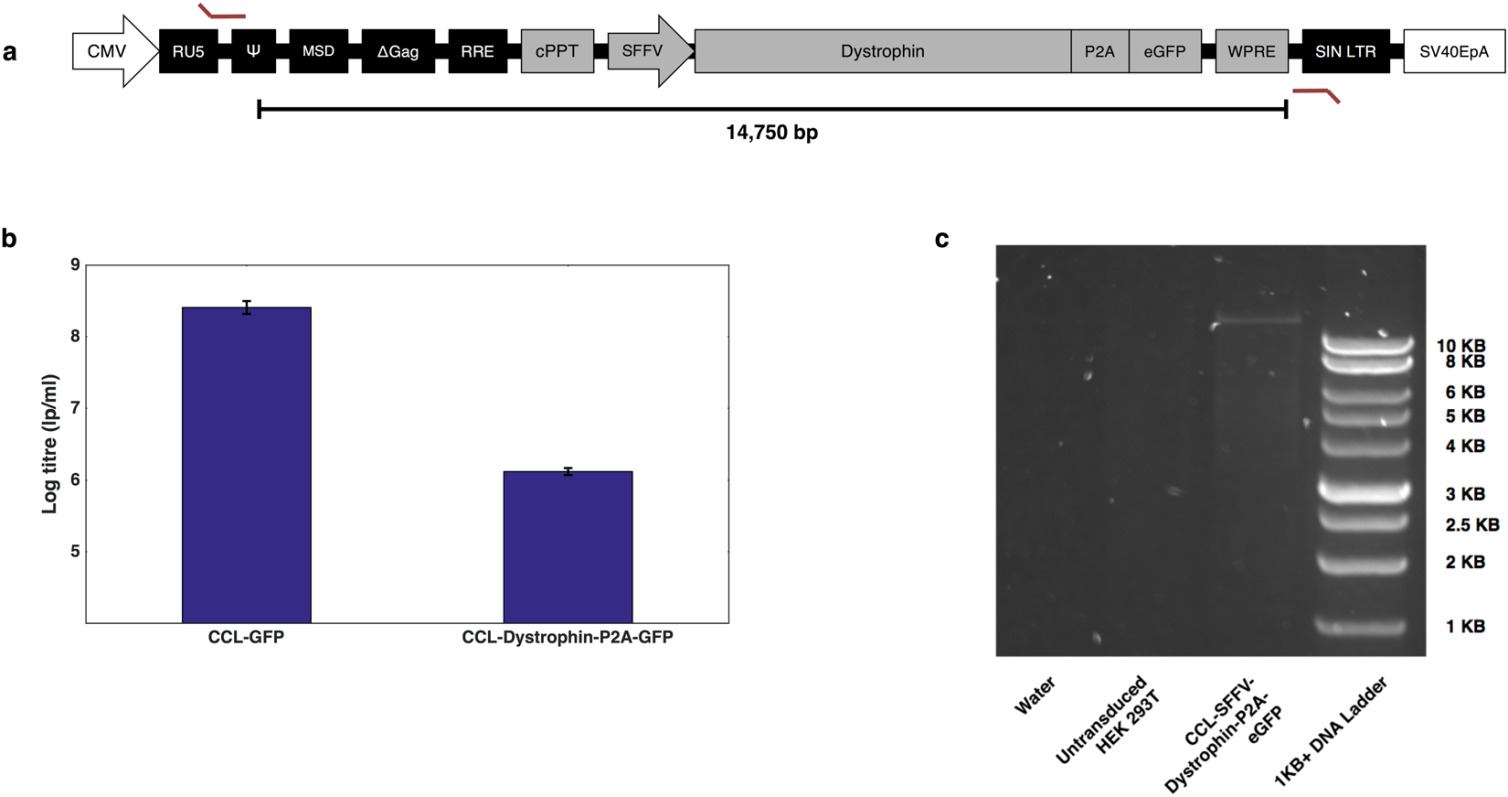
Packaging of full-length dystrophin into a standard lentiviral expression vector. (**a**) Schematic representing the CCL-SFFV-FLAG-Dystrophin-P2A-GFP expression cassette. The locations of primers used for provirus amplification are marked with angled red lines. (**b**) CCL-SFFV-Dystrophin-P2A-GFP was titred by GFP output after HEK 293T transduction. CCL-SFFV-GFP (CCL-GFP) was titred simultaneously to estimate titre-loss from dystrophin payload packaging. This comparison showed that a functional titre >1 × 10^6^ lp/ml can be obtained from a lentivirus containing full-length dystrophin, which is 2 orders of magnitude lower than the CCL-GFP vector. Bars represent mean log titres with standard deviation from the mean. N = 3 for both samples. (**c**) PCR of CCL-SFFV-Dystrophin-P2A-GFP provirus from GFP-sorted HEK 293T genomic DNA. Running samples on a 1% agarose gel reveals a band of more than 10,000 base pairs in the GFP-sorted sample, which is absent from the untreated control. The expected band size for a provirus containing full-length dystrophin is 14,750 base-pairs.

We set out to investigate how efficiently full-length dystrophin could be packaged into a standard lentiviral vector and whether the functional output could be workable for DMD gene therapy. We cloned a lentiviral construct in which full-length dystrophin was under the control of the spleen focus forming virus (SFFV) promoter with an N-terminal FLAG-tag and GFP co-expressed through the P2A cleavage peptide^29^ (Fig. 4a).

The CCL-SFFV-FLAG-Dystrophin-P2A-GFP vector was titred by flow cytometric readout of GFP-positive cells, following HEK 293T transduction. Titration was performed alongside a CCL-SFFV-GFP vector, to gauge the titre-drop resulting from dystrophin packaging. We discovered that full-length dystrophin could be delivered at a titre greater than 1 × 10^6^ lp/ml, approximately 200-fold lower than the CCL-GFP vector (Fig. 4b). The Dystrophin-P2A-GFP positive cells were FACS sorted and genomic DNA was extracted to enable PCR amplification of the integrated provirus (Fig. 4c). Primers targeting the viral LTRs produced an amplicon similar to the expected size (14,750 base pairs). This band was absent from untreated controls. Sequencing of the provirus PCR product matched that of the wild-type dystrophin coding sequence (data not shown), indicating successful full-length dystrophin gene transfer to HEK 293Ts with strong gene transfer fidelity.

**Figure 5:**
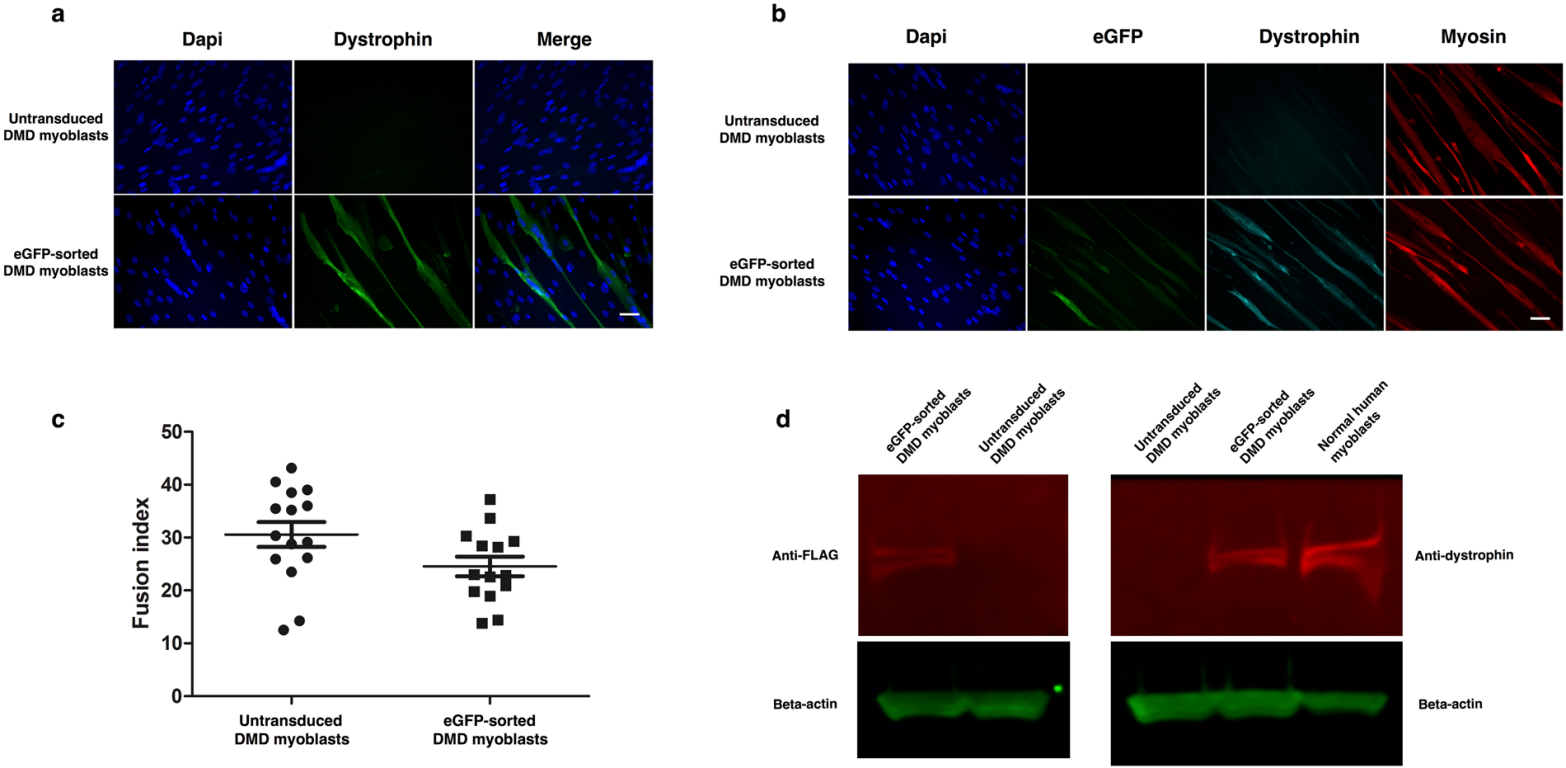
Transduction and differentiation of DMD myoblasts with a lentiviral vector containing full-length dystrophin. (**a**) Staining of differentiated myotubes derived from DMD myoblasts with anti-dystrophin antibody shows successful restoration of dystrophin in a GFP-sorted cell population. This staining was absent from myotubes derived from untransduced controls. Scale bar = 25 μm. (**b**) Staining of differentiated DMD myotubes with anti-GFP, anti-dystrophin or anti-MF20 (myosin marker) antibodies shows successful restoration of dystrophin in differentiated myotubes. Dystrophin staining is located at the sarcolemma of MF20-positive myotubes, demonstrating successful functionality of the dystrophin transgene. This staining pattern is not observed in untransduced controls. Scale bar = 25 μm. (**c**) The fusion index of dystrophin-transduced myoblasts closely resembles that of untransduced control myoblasts. Fusion index was calculated as the proportion of nuclei contained within MF20-positive myotubes, as a percentage of the total nuclei in the image. Data are expressed as median lines with 95% confidence intervals. N = 15 for each data set. (**d**) Western blotting of protein extracts shows expression of full-length dystrophin following lentiviral transduction. Staining with anti-dystrophin (right panel) shows that the GFP-sorted sample contains a dystrophin band matching that of a normal myoblast extract. This band is absent from untreated DMD myoblasts. Staining with anti-FLAG-tag confirms that the lentiviral transgene matches the size of full-length dystrophin and that dystrophin restoration is derived from the exogenous transgene.

To demonstrate *ex vivo* DMD gene therapy with this vector, we transduced human DMD myoblasts at a dose of MOI 0.1 and FACS-purified the GFP-positive cells prior to *in vitro* differentiation. Immunostaining for the dystrophin C-terminus showed strong dystrophin expression on the myotubes derived from GFP-sorted cells, which was absent from the myotubes of untreated DMD controls (Fig. 5a). Subsequent co-staining of dystrophin with anti-GFP and anti-MF20 (myosin marker) confirmed that dystrophin expression was present on the sarcolemma of myotubes that stained positive for myosin (Fig. 5b). This staining pattern was not detected on myotubes derived from untreated controls, which stained positive for myosin, but not dystrophin. The extent of myoblast differentiation was calculated by fusion index on day 7 of differentiation, which showed that the differentiation potential of dystrophin-expressing cells was comparable to untreated cells (Fig. 5c).

Proteins were extracted from the differentiated cells and the samples were compared to healthy controls by western blot to ascertain their size. Staining for the dystrophin C-terminus showed that sorted cells were co-expressing a protein matching the size of full-length dystrophin, whilst staining for the FLAG-tag component confirmed that this protein was derived from the SFFV-FLAG-Dystrophin-P2A-GFP lentiviral transgene (Fig. 5d).

## Discussion

Viral gene transfer is hindered by the packaging limits of clinically applicable viral vectors, which operate with reduced efficiency when delivering transgenes as large as dystrophin. Mini-dystrophin gene therapy and gene editing technologies offer potential solutions to these limitations, although they induce expression of internally deleted dystrophin products that lack full functionality and consequently perform with reduced efficiency^16, 30^. Delivery of full-length dystrophin would allow optimal correction of the DMD phenotype, thus investigations into new technologies are required. *Ex vivo* correction of autologous stem cells with lentiviral vectors provides an effective strategy as it not only ensures that regenerated fibres have the ability to produce dystrophin following transplantation, but also that dystrophin is expressed in stem cells, which has been implicated in regulation of satellite cell polarity and asymmetric division^16^.

In this work we have shown, for the first time, that lentiviral vectors are capable of delivering full-length dystrophin to DMD cells. This offers a significant advancement in the field of DMD gene therapy, given that all functional domains of the vector could be delivered to patient stem cells as an *ex vivo* gene therapy.

Our initial experiments investigating the capacity of standard lentiviruses showed that functional output reduced as the packaging load increased, which is in line with previous studies^2, 18–20^. Our data showed that titres of around 2 × 10^8^ lp/ml can be obtained with a relatively small insert size of 4,400 bp, which would produce a total provirus of approximately 7,096 bp. Given that this payload is smaller than the wild-type HIV-1 genome (~9,600 bp), it seems logical that HIV-1-based lentiviral particles would efficiently package an RNA molecule within this range. Indeed, as the insert size increased beyond the size of wild-type HIV-1, the functional titre dropped, with the largest insert size of 11,099 base pairs returning a titre of 2 × 10^6^ lp/ml. It is unclear whether the limitation was on the efficiency of packaging, reverse-transcription or gene expression. This experiment proved that workable titres were still obtainable with inserts of more than 11,000 base pairs, which suggested that full-length dystrophin delivery could be achievable with lentiviral technology.

We attempted to improve the efficiency of large transgene delivery by designing a novel vector configuration that exploits the recombinogenic nature of HIV-1 reverse-transcriptase. To simplify the system during optimisation, we initially employed a smaller NIGW transgene cassette whilst screening the impact of modifications to vector architecture. The efficiency of NIGW provirus reconstitution in a lentiviral vector containing unmodified elements was comparable with previous reports in which IRES was used as a homologous region for recombination^31, 32^. Interestingly, mutation of core lentiviral *cis* and *trans* elements did not improve the efficiency of this technology. Reverse transcriptase mutants V148I and Q151N and complementary DIS mutations did not increase full-length proviral reconstitution, despite reports that they increase the rate of template-switching^25–28^. This may have been due to impaired infectivity with these variants^33^ and reduced efficiency of provirus synthesis, despite any increase in recombination frequency. From this, we concluded that wild-type HIV-1 components would be the preferred choice for reconstitution of dystrophin sequences.

We further optimised our vector by rearranging the genomes to render lentiviral particles dependent on heterozygous co-packaging for productive reverse-transcription and provide a natural arrangement for strand-transfer to occur within dystrophin sequences, given that obligatory strand-transfer events normally take place at genomic termini^34^. GFP output was detectable in 0.016% of target cells transduced with homozygous vectors, which may have been direct translation of the 3′ dystrophin component, facilitated by the absence of a 5′ leader. Our heterozygous vector configuration produced significantly more GFP-positive cells, although the 0.1% return would require significant improvement for future gene therapy applications. In the NIGW model, recombination may have been facilitated by IRES, which produces a complex secondary structure in RNA molecules, potentially increasing the rate of pausing during reverse-transcription and promoting template-switching^31, 35–39^. Therefore, one potential avenue for improving dystrophin reconstitution could be to incorporate RNA secondary structure into the region of dystrophin homology to force recombination at the intended site.

A nested PCR confirmed that a full-length dystrophin coding sequence had been incorporated into the genomic DNA of cells transduced with the heterozygous vector. However, several smaller PCR products were also obtained from the nested reaction. Sequencing of one band revealed a potential recombination event that would generate a dystrophin variant lacking spectrin repeats 13–19. It is notable that the deleted dystrophin sequence was flanked by adenine-rich sequences, which have been reported to induce reverse transcriptase pausing and promote recombination events^40–42^. A potential strategy for avoiding off-target recombination may be to codon-optimise the transgene to control the frequency of recombination ‘hot-spots’ in dystrophin RNA. This, coupled with incorporation of secondary structure and adenine monobasic runs into the region of homology, could lead to a more efficient and reproducible system. However, we concluded that the template-switching vector could not offer a competitive system for full-length dystrophin gene therapy at this stage.

Previous reports have shown that lentiviruses can package large inserts at the expense of functional titre^2, 18–20^. However, the capacity for lentiviruses to mediate gene transfer of full-length dystrophin has not been demonstrated previously. We have shown that full-length dystrophin can be delivered to target cells via lentiviral technology, even with GFP present in the same transgene cassette, showing that lentiviruses could be used to deliver transgenes larger than dystrophin. The overall yield could limit its clinical translation, although recent advances have been made in maximising lentiviral titres^18, 43, 44^, which may assist in up-scaling this therapeutic strategy.

Our CCL-SFFV-FLAG-Dystrophin-P2A-GFP vector was titred at a yield greater than 1 × 10^6^ lp/ml, which was approximately two orders of magnitude less than a CCL-SFFV-GFP vector. The dystrophin titre was similar to that obtained from our largest stuffer vector, which was also similar in terms of provirus size. This suggests that inserts of approximately 11,000 base pairs can be expected to yield 100-fold lower than standard payloads. However, the mechanism for titre reduction is not clear. There are potentially numerous stages of lentiviral transduction that could be limited by large payloads, such as vector RNA accumulation, reverse-transcription and integration. Successful transduction and integration of our dystrophin lentivirus showed that HIV-1 reverse transcriptase is able to process templates far in excess of its wild-type genome, although it is difficult to pinpoint the cause for titre reduction. Characterising and controlling the individual stages of transduction could be key in improving the performance of lentiviruses carrying large transgenes.

We transduced human DMD myoblasts with our CCL-SFFV-Dystrophin-P2A-GFP vector and sorted cells by GFP positivity before *in vitro* differentiation. Western blotting of protein extracts showed that our lentivirally-expressed dystrophin protein matched the size of wild-type dystrophin. Although dystrophin was occasionally seen on single cells that are myosin negative, the majority of them were present on myosin-positive myotubes, despite the cells being purified based on SFFV-dystrophin-GFP expression (Fig. 5b). This indicated that, although all cells would be expected to contain the dystrophin expression cassette under a constitutive viral promoter, the expressed dystrophin was preferentially translocated to myogenic cells during differentiation, mimicking endogenous dystrophin location. It is possible that post-translational processing of dystrophin and associations with other members of the dystroglycan complex were responsible for this observation^45^.

A primary advantage of lentiviral vectors is their superior payload capacity, which far exceeds that of AAV. At the present time, there is no well-defined cut-off for how much genetic cargo can be packaged into a lentivirus, with previous studies reporting titres with genomes as large as 18,000 base pairs^2^. AAV has a strict capacity of 5,000 base pairs, with genomic truncations impairing delivery of payloads above this limit^3^. It is clear that AAV can be used in many scenarios, although it remains that more than 1,500 human genes would breach the packaging capacity of AAV. As gene therapy continues to expand in translational medicine, it will be necessary to utilise technologies for efficient delivery of large transgenes and we have demonstrated that lentiviruses are able to meet this need.

## Materials and Methods

### Ethics

Human cells were obtained from the MRC Centre for Neuromuscular Diseases Biobank. Tissue sampling was approved by the NHS National Research Ethics Service, Hammersmith and Queen Charlotte’s and Chelsea Research Ethics Committee: Setting up of a Rare Diseases biological samples bank (Biobank) for research to facilitate pharmacological, gene and cell therapy trials in neuromuscular disorders (REC reference number 06/Q0406/33) and the use of cells as a model system to study pathogenesis and therapeutic strategies for Neuromuscular Disorders (REC reference 13/LO/1826), in compliance with national guidelines regarding the use of biopsy tissue for research. All patients or their legal guardians gave written informed consent.

### Generation of plasmid constructs

All transgenes were cloned into either a pRRL or pCCL plasmid backbone^46^ by standard cloning methods and Sanger-sequenced prior to vector production. Lentivirus genome schematics are depicted in Supplementary Figure S1.

The composition of the custom stuffer sequence is outlined in Supplementary Figure S2 with annotations to define the regions packaged into each stuffer-enlarged construct. The stuffer sequence is a contiguous fusion of various transgenes whose extreme termini have been deleted to render them dysfunctional, should any transcription initiate from a cryptic promoter. Potential splice sites and polyadenylation sequences were identified and removed using SplicePort^47^. The stuffer was synthesised by and purchased from GenScript (NJ, USA).

### Lentivirus production and titration

For NIGW viruses, VSV-G-pseudotyped lentiviral vectors were produced by co-transfecting 6 × 10^6^ HEK 293T cells with 2 pmol of the respective pRRL transgene plasmids along with 1 pmol pMDLg.RRE, 0.5 pmol pMD2.G and 0.5 pmol pRSV.REV. For heterozygous viruses, transfections contained 1 pmol of each transgene plasmid. FuGENE®6 (Promega) was used as a transfection reagent at a ratio of 3 μl per 1 μg of DNA.

Dystrophin-containing viruses were produced using a second-generation packaging system^48, 49^. Briefly, 1.5 × 10^7^ HEK 293T cells were transfected with 8 pmol of the respective transgene plasmids, 3.5 pmol of pCMV.dR874 and 2.5 pmol of pMD2.G. For heterozygous viruses, 4 pmol of each transgene plasmid was included to give a total of 8 pmol. DNA mixtures were mixed in 5 ml Opti-MEM® (Life Technologies) and combined with 5 ml Opti-MEM® containing 1 μM polyethylenamine (Sigma). The resulting 10 ml mixture was applied to HEK 293T cells after 20 mins incubation at room temperature.

Virus-containing medium was collected at 48 and 72 hours post-transfection. After each collection, the supernatant was filtered through a cellulose acetate membrane (0.45 μm pore). Lentivirus harvests were combined and stored at 4 °C before ultracentrifugation for 2 h at 90000 × g at 4 °C. Virus pellets were re-suspended in 200 µl of Opti-MEM®.

For NIGW virus titration, 1 × 10^5^ HeLa cells were plated into each well of a 6 well plate and transduced with a range of volumes of the concentrated lentivirus. Seventy-two hours after transduction, HeLa cell genomic DNA was extracted and the proviral titre was calculated by qPCR, as described previously^50^. Dystrophin-containing viruses were titred by p24 ELISA (Clontech 632200) according to the manufacturer’s protocol.

### Transduction of cell lines

For NIGW experiments, HeLa cells were plated onto a flat-bottom 96 well plate at a density of 3 × 10^4^ cells per well. Lentiviruses were introduced 24 hours later at the appropriate MOI in a total volume of 100 μl. For dose-response analysis of NIGW, culture medium was supplemented with neomycin (Geneticin® (Life Technologies)) at a working concentration of 0.4 mg/ml.

For dystrophin reconstitution, HEK 293T cells were plated in 6 well plates at a density of 1 × 10^5^ cells per well. Heterozygous lentivirus preparations were introduced at a dose of 400 ng p24/well, whilst homozygous vectors were co-transduced at 200 ng p24/well for each.

### Flow cytometry detection of transgene reconstitution

Cells were trypsinised and 200 μl of the suspension was added to a round bottom 96-well-plate for analysis in a BD FACSArray™ Bioanalyzer. GFP fluorescence was excited with a 488 nm argon laser. During analysis of cytometry plots, live cell populations were gated by plotting forward-light-scatter versus side-scatter to visualise and isolate the viable population. GFP-positive populations were determined by plotting the emission from the green channel (detected using 530/30 nm band pass filter) against emission from the yellow channel (detected using 575/26 band pass filter), to compensate for auto-fluorescence events. Unless mentioned otherwise, non-transduced populations were used to set the baseline for GFP expression.

During NIGW investigations, homozygous 3′NIGW vectors (without a recognised promoter) expressed low-level GFP, which was presumably driven by an IRES-mediated promoter trap^51, 52^. For this reason, the baseline for GFP was gated against a homozygous control (5′wtDIS + 3′wtDIS sample) to compensate for any IRES-driven expression from unrecombined proviruses. The gated GFP-positive cell populations were used to estimate the amount of reconstituted, full-length proviruses driven by the SFFV promoter.

Where mentioned, GFP-positive cells were sorted on a MoFlo sorting machine.

All FACS data were analysed by FlowJo software version 9.3.1 (©Tree Star, Inc).

### PCR analysis of transduced genomic DNA

Genomic DNA was extracted from cell lines using the DNeasy blood and tissue kit (Qiagen 69504). For PCR detection of full-length NIGW proviruses, 10 ng of genomic DNA was amplified with oligos 5′-GGCAAGTTTGTGGAATTGGT-3′ (targeting Rev-response element) and 5′-AAAGGGAGATCCGACTCGTC-3′ (targeting WPRE) with 2.5 units of *Pfu* polymerase (Thermo Scientific EP0571) with conditions 95 °C for 1 minute; 25 cycles of 95 °C for 30 seconds, 55 °C for 30 seconds, 72 °C for 2 minutes; and a final 72 °C incubation for 5 minutes.

For nested PCR detection of template-switched proviruses, an initial reaction with 400 ng of genomic DNA was performed using oligos 5′-TCAGATGTTTCCAGGCTCCC-3′ (targeting SFFV promoter) and 5′-GAACTTCAGGGTCAGCTTGC-3′ (targeting GFP) with 1 μl of Herculase® II (Agilent 600675) with conditions: 92 °C for 2 minutes; then 10 cycles of 92 °C for 20 seconds, 55 °C for 20 seconds, 68 °C for 6 minutes; followed by 20 cycles of 92 °C for 20 seconds, 55 °C for 20 seconds, 68 °C for 6 minutes plus incremental addition of 20 seconds per cycle; followed by 68 °C for 8 minutes. The nested PCR was then amplified using 1 μl of each initial PCR with oligos 5′-ATCATGGAGCAGAGACTCGG-3′ and 5′-GCTGAGATGCTGGACCAAAG-3′ and 1 μl Herculase® II with conditions 95 °C for 2 minutes; then 20 cycles of 95 °C for 30 seconds, 60 °C for 30 seconds, 68 °C for 12 minutes; followed by 68 °C for 3 minutes.

To determine the size of the SFFV-Dystrophin-P2A-GFP provirus, genomic DNA was PCR-amplified using oligos specific for the lentiviral 5′LTR-PBS junction (5′-AAATCTCTAGCAGTGGCGCCCGAACAG-3′) and the 3′LTR R region (5′-GCACTCAAGGCAAGCTTTATTGAGGCTT-3′)^53^. The PCR was carried out using q5 polymerase (New England Biolabs) with conditions: 98 °C for 30 seconds; 28 cycles of 98 °C for 10 seconds, 72 °C for 1 minute 50 seconds; 72 °C for 2 minutes.

Where necessary, bands of interest were excised and recovered using a QiaQuick gel extraction kit (Qiagen 28704) and subcloned using a Zero Blunt® TOPO® PCR Cloning Kit (Life Technologies 450245) for analysis by Sanger sequencing.

### Maintenance and immunostaining of DMD myoblasts

Human DMD myoblasts were isolated from the left quadriceps of a 3 year old DMD patient with a nonsense mutation in dystrophin exon 42. Myoblasts were maintained in M10 medium (Megacell DMEM medium (Sigma), 10% fetal bovine serum (Invitrogen), 2μM glutamine (Sigma), 1% non-essential amino acids (Sigma), 0.1 mM β- mercaptoethanol (Sigma), 5 ng/ml basic fibroblast growth factor (Peprotech)).

For differentiation, DMD myoblasts were plated onto Matrigel™- coated (BD Bioscience) 8-well chamber slides at a density of 5 × 10^4^ cells per well. Twenty-four hours after plating, culture medium was changed to M2 (Megacell DMEM (Sigma) medium, 2% fetal bovine serum (Invitrogen), 2μM glutamine (Sigma), 1% non-essential amino acids (Sigma), 0.1 mM β- mercaptoethanol (Sigma) and 5 ng/ml basic fibroblast growth factor (Peprotech)) to induce myogenic differentiation.

Cells were fixed with 4% paraformaldehyde after 7 days of differentiation. Fixed cells were blocked with PBS containing 10% goat serum and 0.3% Triton X100 for 1 hour at room temperature. Blocked cells were then incubated with chicken anti-GFP (1:1000, Abcam), rabbit anti-dystrophin (1:1000, Fisher Scientific) and mouse anti-MF20 (1:100, DSHB) antibodies overnight at 4 °C. Alexa-488 conjugated goat anti-chicken IgG (H+L) (1:500, Invitrogen), 594-conjugated goat anti-rabbit IgG (H+L)(1:500, Invitrogen) and 647-conjugated goat anti-mouse IgG2b (1:500, Invitrogen) were introduced for 1 hour at room temperature before adding mounting medium (DAKO product no. CS70330) containing 10 µg/ml 4′,6-diamidino-2-phenylindole (DAPI). Images were captured with a Leica microscope using Metamorph software. The fusion index was quantified as the percentage of nuclei within the MF20 positive myotubes that contain 3 or more nuclei, of the total number of nuclei within the field.

### Western blotting

Protein was extracted from differentiated myoblasts using NCH buffer (4% sodium dodecyl sulphate, 4M urea, 150 mM Tris) diluted 1:1 with RIPA (Radio-Immunoprecipitation Assay) buffer (Sigma) containing a complete protease inhibitor cocktail (1:100, Roche). Samples were collected and boiled for 3 minutes and 30 μl of each sample was run on a NuPAGE Novex 3–8% Tris-Acetate Gel, with constant voltage of 150 V for 1 hour, before transfer to a nitrocellulose membrane at 300 mA for 2 hours. The membrane was blocked in Odyssey blocking solution (LI-COR Biosciences) for 60 min, before incubation with rabbit anti-dystrophin (1:2000, Fisher Scientific) or rabbit anti-FLAG-tag (1:2000, Sigma) and mouse anti-beta-actin (1:4000, Invitrogen) overnight at 4 °C. The membrane was washed with PBS containing 1% Tween 20 (PBST) before incubation with IRDye 680 RD goat anti-rabbit and IRDye 800CW goat anti-mouse 2nd antibodies (1:15000, LI-COR Biosciences) for 1 hour at room temperature. The fluorescent image was acquired by Odyssey Clx infrared imaging system (LI-COR Biosciences) using image studio software 3.1.4.

### Statistical Analysis

FACS data expressed as percentages were compared by either Kruskal-Wallis tests with Dunn’s post-hoc analysis or Mann-Whitney U test. Statistical analyses were carried out using Matlab R2015a or GraphPad Prism software.

## Electronic supplementary material

Supplementary material
