# Supplementary material for: Lentiviral vectors can be used for full-length dystrophin gene therapy
Source: Sci Rep. 2017 Mar 6;7:79. doi: 10.1038/s41598-017-00152-5 (PMC5427806; doi:10.1038/s41598-017-00152-5)
Supplement: Supplementary file 1 — Supplementary material [file 41598_2017_152_MOESM1_ESM.pdf]

Lentiviral vectors can be used for full-length dystrophin gene therapy

Authors

John R. Counsell[1,2,3,4*], Zeinab Asgarian[1†], Jinhong Meng[1†], Veronica Ferrer[2], Conrad A. Vink[3], Steven J. Howe[3], Simon N. Waddington[4,5], Adrian J. Thrasher[3], Francesco Muntoni[1], Jennifer E. Morgan[1] and Olivier Danos[2‡]

Supplementary Material

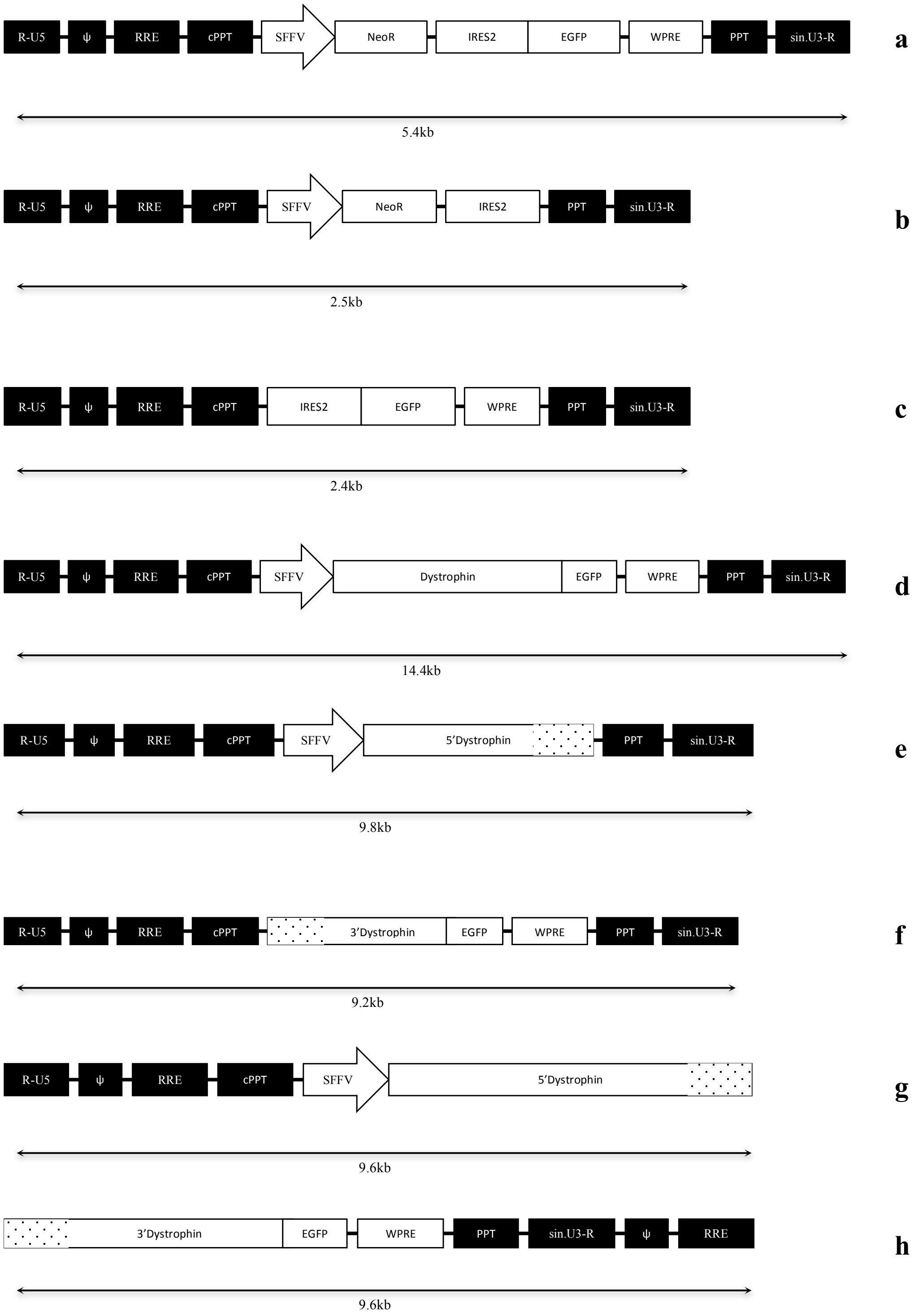

Supplementary Figure S1. Schematics of lentiviral genomes. (**a**) Full-length NIGW. (**b**) 5'NIGW. (**C**) 3'NIGW. (**d**) Full-length dystrophin-GFP. (**e**) SFFV.5'DYS. (**f**) 3'DYS.GFP. (**g**) TS.5'DYS. (**h**) TS.3'DYS. The shaded regions in **e**, **f**, **g** and **h** represent the dystrophin regions of shared homology in which template-switching could occur.

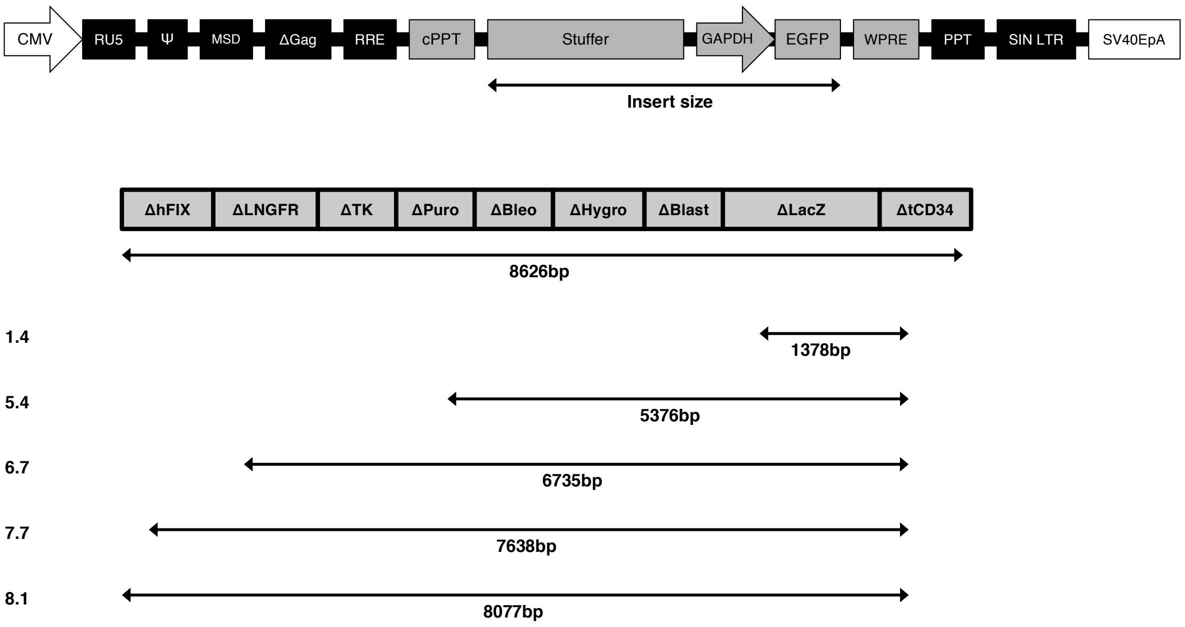

Supplementary Figure S2. Stuffer composition and regions cloned into stuffer constructs. The double-headed arrows beneath the schematic of the full stuffer sequence represent the size and composition of each construct. ΔhFIX – human factor IX cDNA; LNGFR – low-affinity nerve growth factor receptor; TK - tyrosine kinase; Puro – puromycin; Bleo – bleomycin; Hygro – hygromycin; Blast – blasticidin; LacZ - β-galactosidase; tCD34 – truncated human stem cell antigen.

a

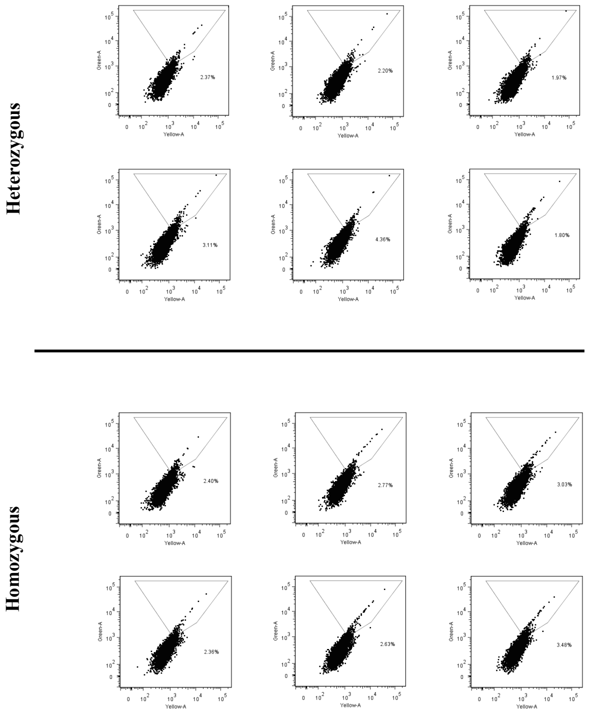
Heterozygous
Homozygous
Green-A
Yellow-A
2.37%
2.20%
1.97%
3.11%
4.36%
1.80%
2.40%
2.77%
3.03%
2.36%
2.63%
3.48%

b

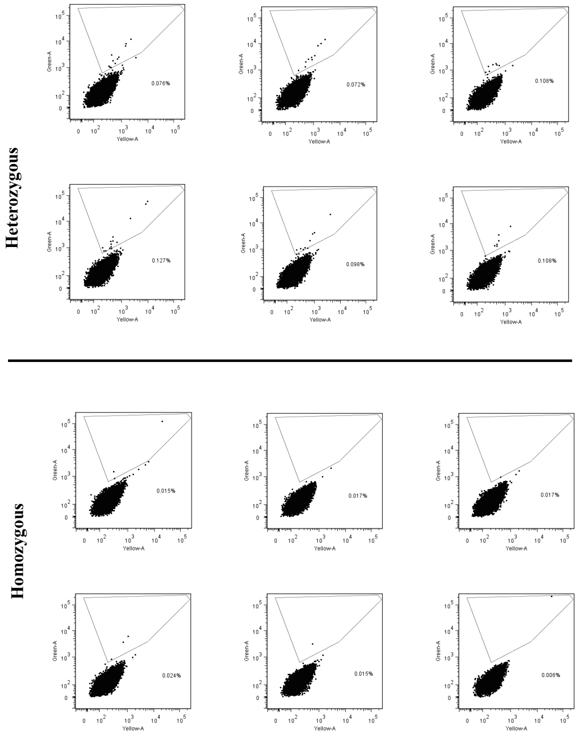
Heterozygous
Homozygous
Green-A
Yellow-A
0.076%
0.072%
0.108%
0.127%
0.098%
0.108%
0.015%
0.017%
0.017%
0.024%
0.015%
0.006%

Supplementary Figure S3. Flow cytometry scatter plots for detection of dystrophin-GFP expression. FACS plots of HEK 293Ts transduced with heterozygous and homozygous lentiviruses: **(a)** initial vector system reported in Figure 3; **(b)** optimised vector system reported in Figure 4. Emission from the yellow channel was used to compensate for auto-fluorescence events. Cells transduced with homozygous vectors show minimal green fluorescence in optimised system, indicating that background GFP expression was markedly reduced compared with the previous vector design. Furthermore, with the optimised system, cells transduced with heterozygous vectors show a significant shift towards green fluorescence.

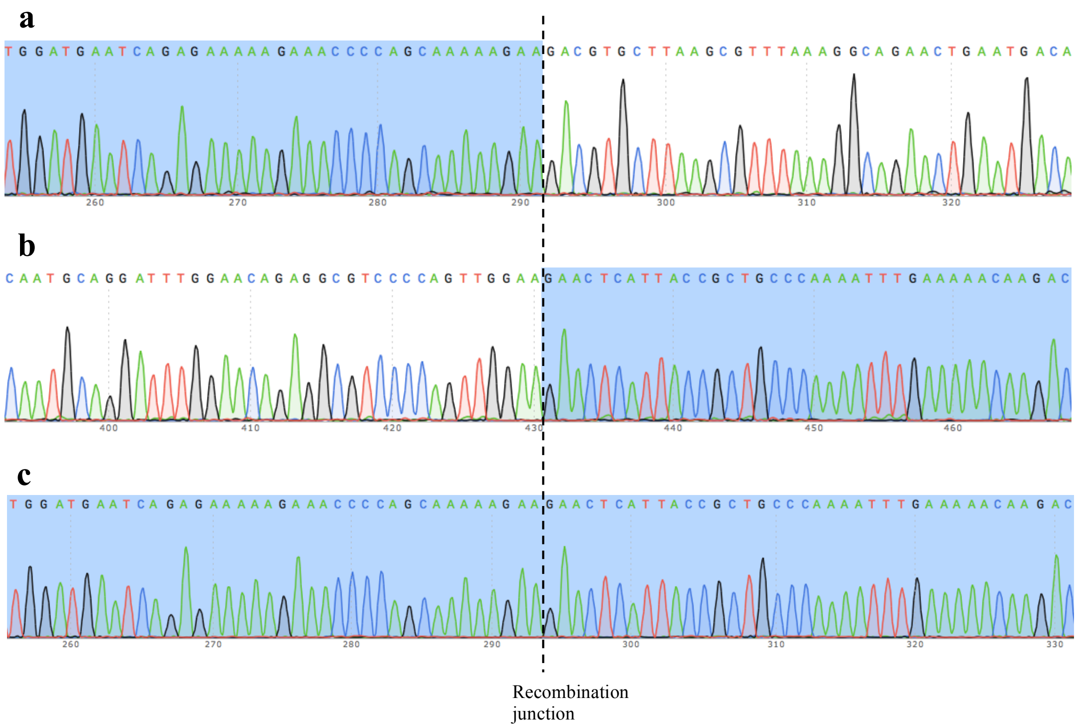

Supplementary Figure S4. Sequence composition of dystrophin PCR products. Sanger sequencing chromatograms for the excised fragments reveal an internally truncated dystrophin sequence. Sequencing of the 6.2kb band through the 5’ **(a)** and 3’ **(b)** regions of dystrophin shows 100% homology to the wild-type sequence. **(c)** Sequencing of the 3.8kb product reveals deletion of dystrophin between bases 5143 and 7588 of the dystrophin coding sequence. The recombination junction is flanked by adenine-rich sequences, suggesting a possible recombination hotspot in dystrophin RNA.
